# Enrichment and Purification of the Bioactive Flavonoids from Flower of *Abelmoschus manihot* (L.) Medic Using Macroporous Resins

**DOI:** 10.3390/molecules23102649

**Published:** 2018-10-16

**Authors:** Zhenzhong Yang, Haitao Tang, Qing Shao, Anna Rita Bilia, Yi Wang, Xiaoping Zhao

**Affiliations:** 1 Pharmaceutical Informatics Institute, College of Pharmaceutical Sciences, Zhejiang University, Hangzhou 310058, China; yangzz@zju.edu.cn (Z.Y.); shaoq@zju.edu.cn (Q.S.); mysky@zju.edu.cn (Y.W.); 2 SZYY Group Pharmaceutical Limited, Taizhou 225500, China; ktang@vip.163.com; 3 Department of Chemistry, University of Florence, via Ugo Schiff 6, Sesto Fiorentino, 50019 Florence, Italy; ar.bilia@unifi.it; 4 School of Basic Medical Sciences, Zhejiang Chinese Medical University, Hangzhou 310053, China

**Keywords:** *Abelmoschus manihot* flowers, macroporous resins, flavonoids

## Abstract

Flower of *Abelmoschus manihot* (FAM) is clinically effective to treat chronic kidney disease (CKD) with a relatively high dosage. To improve the efficacy and the compliance of patients, macroporous resins were adopted to enrich and purify flavonoids from FAM, which are thought to be the major renal protective constituents in FAM. After screening six different kinds of macroporous resins, HPD-100 was selected for its great adsorption and desorption capacity. Then, orthogonal design tests were used to optimize parameters in the processes of impurity removal and flavonoids of FAM desorption on column chromatogram. Moreover, process scale-up was performed, and purification effects maintained after amplification. After purification, the content of seven main flavonoids in the product increased from 8.29% to 51.43%. Protective and anti-inflammatory effects of crude extract and the flavonoid component of FAM after purification were investigated on the adriamycin-damaged HK-2 cells and lipopolysaccharide-stimulated Raw 264.7 cells models. Both bioactivities were improved greatly after purification for these two cell models. Therefore, the purification process had enriched the main bioactive constituents with potential alleviating kidney injury activities. The flavonoid component of FAM is worthy of being developed as an improved remedy for CKD with better patients’ compliance.

## 1. Introduction

*Abelmoschus manihot* (L.) Medic, also known as aibika [1], belongs to the family of *Malvaceae*. This plant is widely distributed throughout India, Sri Lanka, Eastern Indonesia, Papua New Guinea, Vanuatu, Fiji, New Caledonia, Northern Australia, and China [2,3]. In Papua New Guinea, Eastern Indonesia, and the South Pacific Islands, *A. manihot* is a popular vegetable, which is reported to be of high nutritional value [1,3]. In China, various functional foods have been developed from roots, stems, and leaves of *A. manihot* [4,5]. Flowers of *A. manihot* (FAM), the main medicinal part of this plant, have been used in clinical practice to treat chronic kidney disease (CKD), inflammatory diseases, oral ulcers, and burns in China for hundreds of years [5,6].

Huangkui capsule (HKC), a single plant drug extracted from FAM, has been proved to be clinically effective in reducing proteinuria and protecting kidney function in CKD [7,8,9,10]. However, the dosage of HKC was relatively high, at 7.5 g/d for an adult, which might influence the patients’ compliance. Flavonoids are thought to be the major bioactive components in *A. manihot* responsible for protecting renal function [11,12,13]. Therefore, attempts were made to enrich and purify the flavonoids to reduce the clinical dosage of the relevant remedy derived from *A. manihot*. Several efforts have been made to improve the purity of flavonoids through extraction and purification processes [14,15,16,17]. However, a more effective and robust purification strategy is needed for industrial development. Herein, macroporous resins, as powerful materials for enrichment and purification [18,19,20], were adopted to purify the bioactive components from the extract of *A. manihot* in this study. Two bioassay models, i.e., adriamycin-damaged renal proximal tubular epithelial cells and lipopolysaccharide (LPS)-stimulated macrophage cells, were employed to evaluate the improvement in bioactivity after purification.

## 2. Results and Discussion

### 2.1. Screening of Macroporous Resins

**Figure 1:**
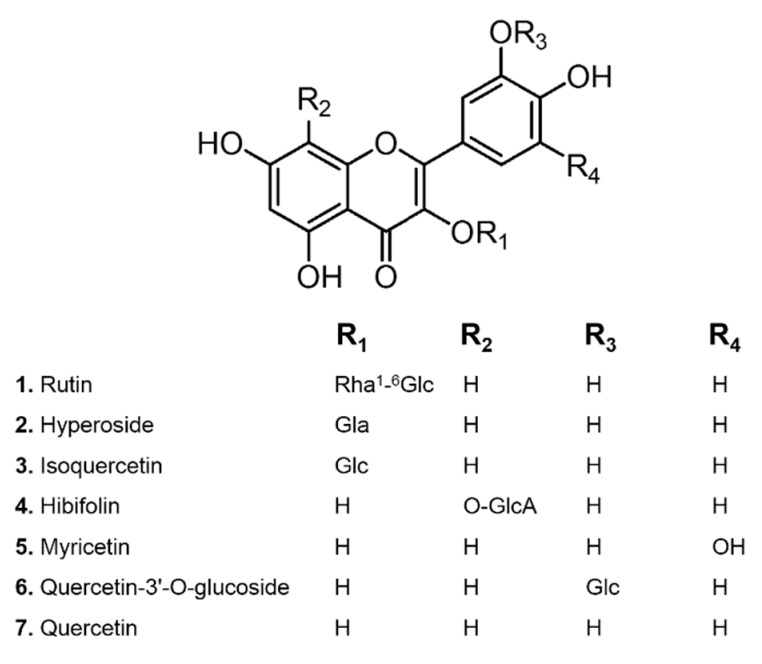
The chemical structures of flavonoids from *A. manihot*.

**Figure 2:**
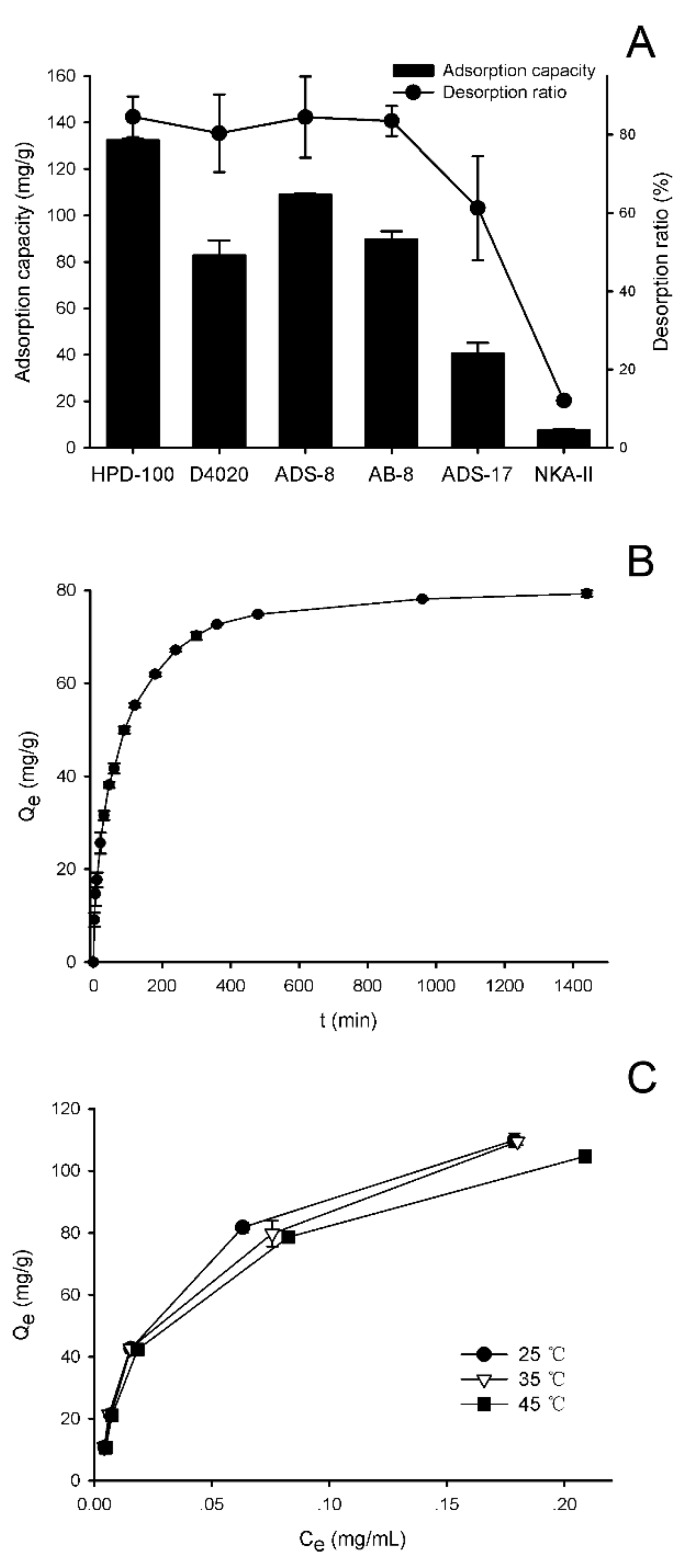
Adsorption and desorption of flower of *Abelmoschus manihot* (FAM) flavonoids on macroporous resins. (**A**) Static adsorption capacity and desorption ratio of different macroporous resins; (**B**) Static adsorption kinetics of FAM flavonoids on HPD-100 resins; (**C**) Adsorption isotherms for FAM flavonoids on HPD-100 resins at different temperatures.

To effectively enrich and purify the flavonoids, the adsorption and desorption capacities of FAM flavonoids on six different kinds of macroporous resins, i.e., HPD-100, D4020, ADS-8, AB-8, ADS-17, and NKA-II, were investigated. The physical properties of these resins used were summarized in Table 1. Contents of seven main flavonoids in FAM (structures shown in Figure 1) was determined using a UPLC method to indicate the performance. As shown in Figure 2A, HPD-100 demonstrated the highest adsorption capacity (132 mg/g dry resin), and other non-polar or weak-polar resins, i.e., D4020, ADS-8, and AB-8, also exhibited considerable adsorption capacities. However, the adsorption capacity of the moderately-polar resins ADS-17 was significantly lower than the non-polar or weak-polar ones, while the polar resin NKA-II showed the lowest adsorption capacity.

The desorption ratios of FAM flavonoids for non-polar, weak-polar, and moderately-polar resins in 60% ethanol solution were all above 60%. Among these resins, HPD-100 resin exhibited the highest desorption capacity. Therefore, HPD-100 resin was selected for further investigation.

### 2.2. Adsorption Kinetics of FAM Flavonoids on HPD-100 Resins

The adsorption kinetic curve for HPD-100 resin was presented in Figure 2B. The adsorption capacity of HPD-100 resin showed a linear and rapid increase over the first 90 min. Then, the adsorption capacity of HPD-100 resin increased more slowly and reached the adsorption equilibrium at 360 min.

### 2.3. Adsorption Isotherms of FAM Flavonoids on HPD-100 Resins

The adsorption isotherms of HPD-100 resin were constructed at 25 °C, 35 °C, and 45 °C, and the results were presented in Figure 2C.

The adsorption capacity of HPD-100 resin increased rapidly at low concentrations of FAM flavonoids, and then slowly approached a saturation plateau. At the same initial concentration of FAM flavonoids, the adsorption capacities decreased when the temperature increased, which indicated that the adsorption process was an exothermic process. Thus, 25 °C was chosen for use in further experiments.

Two isotherm models, i.e., the Langmuir and Freundlich equations, were used to understand the adsorption mechanism of the adsorbate to the adsorbent. The Langmuir and Freundlich parameters of FAM flavonoids on HPD-100 resins were summarized in Table 2. The Langmuir model, with good correlation coefficients (*R*^2^ > 0.98) at different temperatures, seemed to be a good model for reflecting the adsorption equilibriums of FAM flavonoids on HPD-100 resins, which indicated that the adsorption behavior was monolayer solid–liquid adsorption.

### 2.4. Optimization of Conditions for Purification of FAM Flavonoids by HPD-100 Resins

The glass column, wet-packed with HPD-100 resins, was used to purify the extract from FAM. The process of purification included two steps, removing impurity and desorption, both of which played important roles in the process of purification.

In this study, an orthogonal design was employed to optimize the parameters in the processes of removing impurity and desorption of FAM flavonoids. The selected factors and their levels are listed in Table 3 and Table 4. The results of the orthogonal tests are presented in Table 5 and Table 6. Contents of seven main flavonoids in FAM were determined by a UPLC method, which was used to indicate the performance of both processes. The value of ki was used to judge the optimal level of each factor, while R was used to indicate the importance of the factors. In the process of removing impurity, the optimized conditions for removing impurity were A3/B3/C3, and the importance order of the factors was A > B > C. The ethanol concentration of the solvent for removing impurity was the most pivotal factor, and could influence the contents of the seven main flavonoids in FAM easily. The volume of the solvent for removing impurity was another important factor. A sufficient solvent volume could flush the impurity adequately. The concentrations of the main flavonoids in eluent scarcely varied while the flow rate increased, which indicated the flow rate didn’t have much influence on the performance. These optimized parameters of the impurity removing process were adopted in further experiments.

In the process of desorption of the FAM flavonoids from HPD-100 resins, the optimized conditions for desorption were D1/E3/F3, and the importance order of the factors was E > F > D, which was quite different from that of the impurity removing process. A sufficient volume of solvent was needed to flush these flavonoids down from the HPD-100 resins, while the ethanol concentration of the solvent and its flow rate didn’t play important roles in this process. In further experiments, these optimized parameters of the desorption process were employed.

The validation experiments were performed using the optimized conditions in the processes of removing impurity and desorption of FAM flavonoids. The content of the seven main flavonoids in FAM was 51.6%, which indicated the great purification effects and good repeatability of the optimized purification processes.

### 2.5. Process Scale-Up

**Figure 3:**
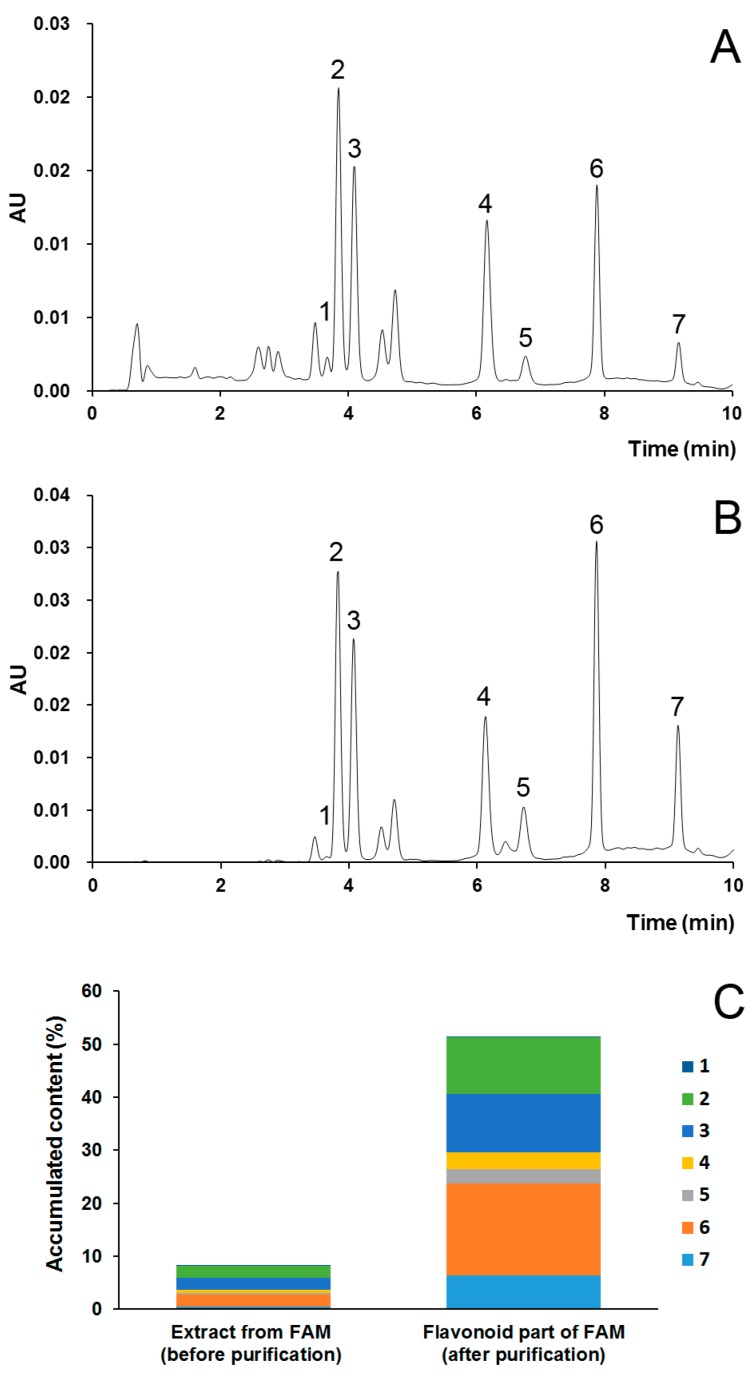
UPLC chromatograms of crude extract from FAM (before purification, (**A**)) and flavonoid part of FAM (after purification, (**B** ), and the accumulated content of the seven main flavonoids (**C**). 1. Rutin, 2. Hyperoside, 3. Isoquercetin, 4. Hibifolin, 5. Myricetin, 6. Quercetin-3′-*O*-glucoside, 7. Quercetin.

For the further industrial applications, experiments were performed on a larger scale on a column (9 cm × 45 cm), with greater than eleven times amplification. UPLC chromatograms of extract from FAM (before purification) and flavonoid part of FAM (after purification) are presented in Figure 3. The content of the seven main flavonoids in the purified part of flavonoids was 514.3 mg/g (51.43%), while that of the crude extract from FAM before purification was 82.9 mg/g (8.29%). The total flavonoid content was also determined with the colorimetric method using hyperoside as a reference standard. After purification, the total flavonoid content was 705.6 mg/g (70.56%), while that of the crude FAM extract before purification was 202.5 mg/g (20.25%). These results indicated that good purification effects were maintained upon scale-up of the process, which demonstrated that this purification strategy was promising for further industrial development. 

### 2.6. Protective Effects on the Adriamycin-Damaged HK-2 Cells

**Figure 4:**
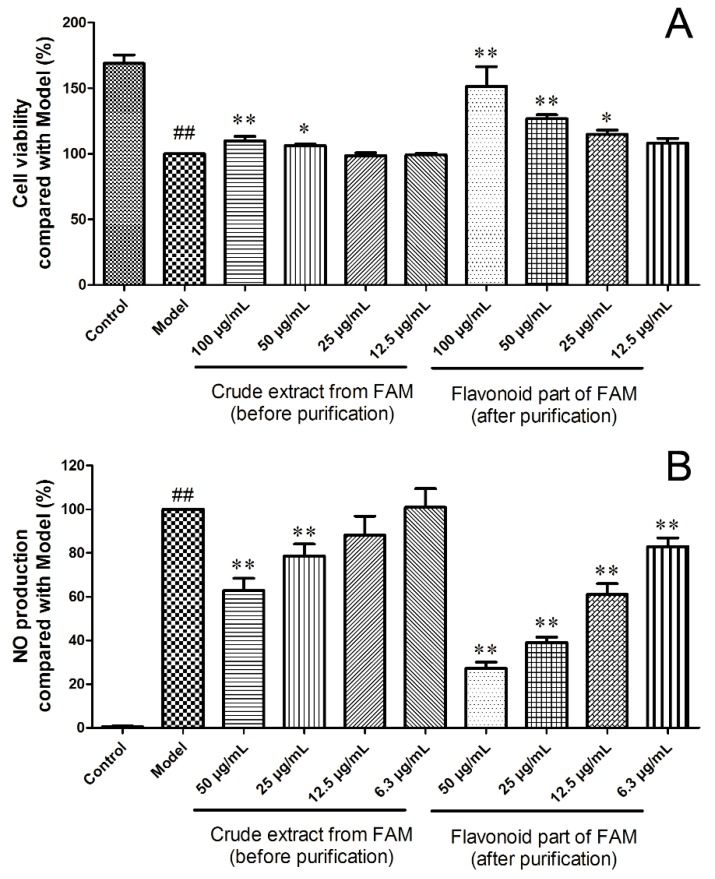
Potential nephroprotective (**A**) and anti-inflammatory (**B**) effects of crude extract from FAM (before purification) and the flavonoid part of FAM (after purification). ^##^ *p* < 0.01 vs. Control, * *p* < 0.05 vs. Model, ** *p* < 0.01 vs. Model.

To compare the protective effects before and after purification, adriamycin-damaged HK-2 cells were treated with relevant samples at concentrations without cytotoxicity. In the model group, HK-2 cells were significantly damaged by adriamycin, compared with the control group, as shown in Figure 4A. The extract from FAM before purification showed a potential nephroprotective effect at the concentration from 50 to 100 μg/mL, which was consistent with our previous report [21]. The potential nephroprotective effect of the flavonoid component of FAM after purification improved greatly, which ameliorated the adriamycin-induced damage when the concentration was above 25 μg/mL. Additionally, the protective effect on adriamycin-induced damage of the flavonoid component of FAM at 25 μg/mL was comparable to that of the extract before purification at 100 μg/mL. Thus, the purification process had enriched the main bioactive constituents with potential nephroprotective effect.

### 2.7. Anti-Inflammatory Effects on the LPS-Stimulated Raw 264.7 Cells

An increased number of macrophages are found in a diseased kidney, which plays an important role in inflammation and renal injury in various acute and chronic kidney diseases [22]. The reduction of inflammation is regarded as an effective therapeutic strategy against renal injury [23]. Nitric oxide (NO), as an important inflammatory mediator, is released by activated macrophages [24,25]. The NO production in LPS-stimulated Raw 264.7 cells was determined to compare the anti-inflammatory effects before and after purification at concentrations with no cytotoxicity. As shown in Figure 4B, the NO amount in the model group increased significantly compared to the control group. Raw 264.7 cells were activated by LPS and treated with extract from FAM before or after purification. In both treatments, the NO production in LPS-stimulated Raw 264.7 cells decreased in concentration-dependent manners. The extract from FAM before purification showed an anti-inflammatory effect at the concentration from 25 to 50 μg/mL. After purification, the anti-inflammatory effect of the flavonoid part of FAM was greatly enhanced, exhibiting significant anti-inflammatory effects with concentrations above 6.3 μg/mL. The flavonoid part of FAM at a concentration of 6.3 μg/mL presented a similar anti-inflammatory effect to the extract before purification at 25 μg/mL for this cell model. The main constituents responsible for the anti-inflammatory effects were enriched through the purification process. Further cellular and animal experiments are needed to confirm these effects.

## 3. Materials and Methods

### 3.1. Materials and Chemicals

Macroporous resins (HPD-100, D4020, ADS-8, AB-8, ADS-17 and NKA-II) were purchased from Cangzhou Bon Adsorber Technology Co., Ltd. (Cangzhou, China). High-performance liquid chromatography (HPLC) grade acetonitrile was purchased from Merck (Darmstadt, Germany). HPLC grade formic acid was purchased from Roe Scientific Inc. Water was purified by the Milli-Q system (Millipore, Bedford, MA, USA). Hyperoside was purchased from Ronghe Pharmaceutical Technology Development Co., Ltd. (Shanghai, China). FAM was collected in October 2015 from Jiangsu Province, China. The materials were authenticated by Qiufeng Song et al. Extract from FAM was obtained from Suzhong Pharmaceutical Group Co., Ltd. (Taizhou, China).

Dulbecco’s modified Eagle’s medium (DMEM) and DMEM/F-12 were purchased from Corning (Manassas, VA, USA). Fetal bovine serum (FBS), penicillin, streptomycin, and 0.25% Trypsin-EDTA were purchased from Gibco (Grand Island, NY, USA). 3-(4,5-dimethyl-thiazol-2-yl)-2,5-diphenyl tetrazolium bromide (MTT) and LPS were purchased from Sigma (St. Louis, MO, USA). Adriamycin was obtained from Shanghai Aladdin Biochemical Technology Co., Ltd. (Shanghai, China). An NO assay kit was purchased from Beyotime Institute of Biotechnology (Nanjing, China).

### 3.2. Pretreatment of Macroporous Resins

The resins were pretreated by soaking in 95% ethanol overnight, and then washed with distilled water thoroughly to remove ethanol completely.

### 3.3. Static Adsorption and Desorption Tests

The static adsorption and desorption tests for the enrichment and purification of flavonoids using the macroporous adsorption resins were performed as follows: 2 g of the pretreated resin was added to a conical flask, followed by 50 mL of extract of FAM with a concentration of 25 mg/mL. These flasks were sealed with stoppers and placed in a thermostatic oscillator. The whole process of adsorption was performed at 25 °C with a shaking speed of 150 rpm for 12 h. After adsorption, resins were washed three times with distilled water. For desorption, 50 mL of 60% ethanol solution was added to the flasks. The flasks were shaken at 25 °C with a shaking speed of 150 rpm for 12 h to reach desorption equilibrium.

After adsorption and desorption, the solutions were centrifuged at 10,000 rpm for 10 min, and the supernatants were taken for determining the contents of flavonoids.

The adsorption capacity of resins, Q*_e_* (mg/g), was calculated according to Equation (1):

(1) $$\;Q_{e}=\frac{\left(C_{0}-C_{e}\right)V_{i}}{W}\;$$

where Q*_e_* is the adsorption capacity at the adsorption equilibrium (mg/g dry resin), *C*_0_ and *C_e_* are the initial and equilibrium concentrations (mg/mL) of flavonoids in the solution, respectively, *V_i_* is the volume of the initial sample solution (mL), and, *W* is the weight of dry resin (g).

The desorption ratio (%) were calculated according to Equation (2):

(2) $$\;R_{d}\left(\%\right)=\frac{C_{d}\times V_{d}}{\left(C_{0}-C_{e}\right)V_{i}}\times 100\%\;$$

where *C_d_* is the concentration of flavonoids in the desorption solution (mg /mL), *V_d_* is the volume of the desorption solution (mL), and *C*_0_, *C_e_,* and *V_i_* are the same as described above.

### 3.4. Adsorption Kinetics of Flavonoids from FAM on Macroporous Resin

The pretreated resin (3.0 g) was accurately weighed and transferred into a conical flask. The extract of FAM (50 mL with a concentration of 25 mg/mL) was added into the conical flasks. These flasks were sealed with stoppers, placed in a thermostatic oscillator, and shaken at speed of 150 rpm for 24 h at 25 °C. 

Liquid extract (200 μL) was withdrawn at each time point of 0, 2, 5, 10, 20, 30, 45, 60, 90, 120, 180, 240, 300, 360, 480, 960, and 1440 min. The contents of the seven main flavonoids were determined.

### 3.5. Adsorption Isotherms of Flavonoids from FAM on Macroporous Resin

The pretreated resin (1.0 g) was added to a conical flask. FAM extract (50 mL) with different concentrations were added into the conical flasks. The sealed flasks were shaken at speed of 150 rpm for 12 h at 25 °C, 35 °C, or 45 °C in a thermostatic oscillator. The contents of the seven main flavonoids in the supernatants were determined. 

The Langmuir and Freundlich models were employed to reveal the fitting and describe the adsorption property of the resin. The Langmuir model can be expressed by Equation (3):

(3) $$\;Q_{e}=\frac{q_{m}K_{l}C_{e}}{1+K_{l}C_{e}}\;$$

where Q*_e_* (mg/g) is the adsorption capacity at equilibrium; *q_m_* (mg/g) is the maximum adsorption capacity of the adsorbent; *K_l_* is the Langmuir constant; and *C_e_* (mg/mL) is the concentration of flavonoids at equilibrium. 

The Freundlich model can be expressed by Equation (4):

(4) $$\;Q_{e}=K_{f}C_{e}^{1/n}\;$$

where *K_f_* is the Freundlich constant; and 1/*n* is an empirical constant related to the adsorption intensity of the resin.

### 3.6. Optimization of Conditions for Removing Impurity by HPD-100 Resin

A column chromatogram method was adopted for removing impurity from extract of FAM. HPD-100 resin was wet-packed in the column (4 cm × 20 cm), while different concentrations of ethanol solution were used to removing impurity and desorption.

An orthogonal design, L_9_(3^4^), was employed to optimize the conditions for removing impurity by HPD-100 resin. The concentration of ethanol in solvent, the volume of the solvent, and the flow rate of the eluent were selected for optimization. The ethanol concentration of the eluent (%, *v*/*v*) was coded as A, the volume of the eluent was coded as B, and the flow rate was coded as C. All samples used in the orthogonal experiment were pre-flushed with four bed volumes (BV) of water at a flow rate of 40 mL/(cm^2^·h). After removing the impurity, ethanol solvent with a concentration of 70% was used to desorb FAM flavonoids from HPD-100 resin. Finally, two BV of 95% ethanol solvent were used to refresh the column.

### 3.7. Optimization of Conditions for Desorption of FAM Flavonoids from HPD-100 Resin

After the optimization of conditions for removing the impurity, the parameters for the desorption of FAM flavonoids from HPD-100 resin were also optimized using an orthogonal design. Similarly, the concentration of ethanol in eluent for desorption, the volume of the eluent, and the flow rate of the eluent were variables selected for optimization. The ethanol concentration of the eluent for desorption (%, *v*/*v*), the volume of the eluent, and the flow rate were coded as D, E, and F, respectively. All samples used in the orthogonal experiment were pre-flushed with four BV of water at a flow rate of 40 mL/(cm^2^·h), and then optimized conditions were adopted to remove the impurity. Afterward, different desorption conditions were performed to desorb FAM flavonoids from HPD-100 resin.

### 3.8. Determination of the Total Flavonoid Content

The total flavonoid content was determined using a colorimetric method described previously [26,27] with slight changes. Briefly, sample solution was added to a volumetric flask, followed by the addition of 5% (*m*/*v*) NaNO_2_ solution (1 mL). After 6 min, 1 mL of 10% (*m*/*v*) Al(NO_3_)_3_ solution was added and allowed to stand for another 6 min before 10 mL of 1.0 M NaOH was added. After 15 min, the absorbance was measured against the blank at 500 nm using an ultraviolet-visible spectrophotometer (Cary 60, Agilent Technologies, Penang, Malaysia) in comparison with the standards prepared similarly with the known hyperoside concentrations.

### 3.9. Determination of Flavonoids by a Single Reference Standard

The concentration of the main flavonoids in the extracts was determined according to the method described previously [28]. Briefly, the separation was performed on an ACQUITY UPLC BEH C18 column (2.1 mm × 100 mm, 1.7 μm) at a solvent flow rate of 0.4 mL/min at 35 °C. Acidified water (0.05% formic acid) and acetonitrile were used as mobile phases A and B, respectively. The solvent gradient adopted was as follows: 0–2 min, 12–15% B; 2–5 min, 15–16% B; 5–6 min, 16–17% B; 6–9 min, 17–30% B; 9–11 min, 30–80% B; 11–12 min, 80–95% B. The detection wavelength was 360 nm and the injection volume was 1 μL. Hyperoside was selected as the internal reference standard because it was easy to obtain, low cost, and stable. The conversion factors (Fx) of the rest six flavonoids reported were adopted [28].

### 3.10. Cell Culture

Human renal proximal tubular epithelial cell line HK-2 cells and murine macrophage cell line Raw 264.7 cells were obtained from the Cell Bank of Type Culture Collection of the Chinese Academy of Sciences (Shanghai, China). HK-2 cells were cultured in DMEM/F-12 containing 10% FBS and penicillin/streptomycin. Raw 264.7 cells were grown in DMEM containing 10% inactivated FBS and penicillin/streptomycin. Both cultures were maintained in a humidified atmosphere of 5% CO_2_ at 37 °C.

### 3.11. Cell Viability Assay

Cell viability was determined by MTT assay. The HK-2 cells and the Raw 264.7 cells were seeded at 5 × 10^3^ cells/well and 3 × 10^3^ cells/well in 96-well plates, respectively. After 24-h incubation, the cells were treated with different concentrations of tested samples for another 24 h. At the end, 100 μL of MTT solution (0.5 mg/mL) was added to the HK-2 cells. After the wells were incubated for another 4 h at 37 °C, the medium was removed, and the synthesized formazan crystals were dissolved in 100 μL of DMSO. Finally, absorbance was measured at 580 nm using an infinite M1000 microplate reader instrument (Tecan, Grodig, Austria). Data were calculated as a percentage of MTT compared to control cells.

### 3.12. Cell Viability on the Adriamycin-Damaged HK-2 Cells

The HK-2 cells were seeded at 5 × 10^3^ cells/well in 96-well plates and incubated at 37 °C. After 24-h incubation, the cells were damaged by 10 μM of adriamycin for 24 h, and different concentrations of tested samples were added simultaneously. The potential nephroprotective effect was evaluated using the MTT-based method as used in the cell viability assay. Data were calculated as a percentage of MTT compared to Model group.

### 3.13. NO production on the LPS-Stimulated Raw 264.7 Cells

The Raw 264.7 cells were seeded at a density of 2 × 10^4^ cells/well in 96-well plates and incubated at 37 °C. After 24-h incubation, the cells were activated by 200 ng/mL of LPS for 24 h, and samples with different concentrations were added. After incubation, the supernatant of the cell culture was collected to mix with the Griess reagent. The absorbance was measured at 550 nm using an Infinite M1000 microplate reader instrument (Tecan, Grodig, Austria).

## 4. Conclusions

In this study, HPD-100 resin was selected for the enrichment and purification of flavonoids of FAM through static adsorption and desorption experiments. The processes of removing impurity and desorption on column chromatogram were optimized by orthogonal design tests, and the conditions proved to be robust upon scale-up of the process. Protective and anti-inflammatory effects of the flavonoid part of FAM after purification were stronger than that of crude extract from FAM before purification on two cell models, indicating that the main bioactive constituents with potential alleviate kidney injury activities were enriched. Further investigations may be performed in cellular and animal models to confirm these effects in the future. The development of the flavonoid part of FAM as an improved remedy for CKD with better compliance of patients is promising.

## Figures and Tables

**Table 1:** Specifications of the six macroporous resins.

Macroporous Resin	Polarity	Material	Particle Size (mm)	Surface Area (m^2^/g)	Average Pore Diameter (Å)
HPD-100	Non-polar	Polystyrene	0.3~1.2	650~700	85~90
D4020	Non-polar	Polystyrene	0.30~1.25	540~580	100~105
ADS-8	Non-polar	Polystyrene	0.30~1.25	450~500	120~160
AB-8	Weak-polar	Polystyrene	0.30~1.25	480~520	130~140
ADS-17	Moderately-polar	Polystyrene	0.30~1.25	90~150	250~300
NKA-II	Polar	Polystyrene	0.30~1.25	160~200	145~155

**Table 2:** Adsorption isotherms parameters of FAM flavonoids on HPD-100 resins at different temperatures.

Temperature (°C)	Langmuir Model			Freundlich Model		
	*q_m_*	*K_l_*	*R* ^2^	1/*n*	*K_f_*	*R* ^2^
25	131.3420	0.0362	0.9948	0.4575	252.7008	0.9441
35	125.8467	0.0346	0.9826	0.4603	247.3965	0.9690
45	121.3182	0.0385	0.9913	0.4482	219.4200	0.9586

**Table 3:** Factors and levels of conditions for removing impurity by HPD-100 resin.

Level	Factor		
	A (Ethanol Concentration)	B (Solvent Volume)	C (Flow Rate)
1	10%	2 BV	20 mL/(cm^2^·h)
2	20%	3 BV	40 mL/(cm^2^·h)
3	30%	4 BV	60 mL/(cm^2^·h)

**Table 4:** Factors and levels of conditions for desorption of FAM flavonoids from HPD-100 resin.

Level	Factor		
	D (Ethanol Concentration)	E (Solvent Volume)	F (Flow Rate)
1	50%	2 BV	20 mL/(cm^2^·h)
2	60%	4 BV	40 mL/(cm^2^·h)
3	70%	6 BV	60 mL/(cm^2^·h)

**Table 5:** Experimental results of the orthogonal test for removing impurity by HPD-100 resins.

Trial No.	A (Ethanol Concentration)	B (Solvent Volume)	C (Flow Rate)	Contents of 7 Main Flavonoids/%
1	10%	2 BV	20 mL/(cm^2^·h)	22.44
2	20%	2 BV	40 mL/(cm^2^·h)	29.77
3	30%	2 BV	60 mL/(cm^2^·h)	40.61
4	10%	3 BV	40 mL/(cm^2^·h)	27.81
5	20%	3 BV	60 mL/(cm^2^·h)	33.91
6	30%	3 BV	20 mL/(cm^2^·h)	44.06
7	10%	4 BV	60 mL/(cm^2^·h)	27.70
8	20%	4 BV	20 mL/(cm^2^·h)	34.14
9	30%	4 BV	40 mL/(cm^2^·h)	44.20
k1	25.98	30.94	33.55	
k2	32.61	35.26	33.93	
k3	42.96	35.35	34.07	
R	16.97	4.40	0.53	

Note: ki is the mean contents of seven main flavonoids with the corresponding level of each factor, R is the range of ki of each factor.

**Table 6:** Experimental results of the orthogonal test for desorption of FAM flavonoids from HPD-100 resins.

Trial No.	D (Ethanol Concentration)	E (Solvent Volume)	F (Flow Rate)	Contents of 7 Main Flavonoids/%
1	50%	2 BV	20 mL/(cm^2^·h)	43.23
2	60%	2 BV	40 mL/(cm^2^·h)	43.32
3	70%	2 BV	60 mL/(cm^2^·h)	44.79
4	50%	4 BV	40 mL/(cm^2^·h)	50.37
5	60%	4 BV	60 mL/(cm^2^·h)	49.37
6	70%	4 BV	20 mL/(cm^2^·h)	51.02
7	50%	6 BV	60 mL/(cm^2^·h)	53.72
8	60%	6 BV	20 mL/(cm^2^·h)	51.51
9	70%	6 BV	40 mL/(cm^2^·h)	48.52
k1	49.11	43.78	48.59	
k2	48.07	50.25	47.40	
k3	48.11	51.25	49.29	
R	1.04	7.46	1.89	

Note: ki is the mean contents of seven main flavonoids with the corresponding level of each factor, R is the range of ki of each factor.
